# Engineering Botulinum Neurotoxins for Enhanced Therapeutic Applications and Vaccine Development

**DOI:** 10.3390/toxins13010001

**Published:** 2020-12-22

**Authors:** Christine Rasetti-Escargueil, Michel R. Popoff

**Affiliations:** Toxines Bacteriennes, Institut Pasteur, 75724 Paris, France; popoff2m@gmail.com

**Keywords:** botulinum neurotoxin, *Clostridium botulinum*, therapeutic application, recombinant toxin, toxin engineering

## Abstract

Botulinum neurotoxins (BoNTs) show increasing therapeutic applications ranging from treatment of locally paralyzed muscles to cosmetic benefits. At first, in the 1970s, BoNT was used for the treatment of strabismus, however, nowadays, BoNT has multiple medical applications including the treatment of muscle hyperactivity such as strabismus, dystonia, movement disorders, hemifacial spasm, essential tremor, tics, cervical dystonia, cerebral palsy, as well as secretory disorders (hyperhidrosis, sialorrhea) and pain syndromes such as chronic migraine. This review summarizes current knowledge related to engineering of botulinum toxins, with particular emphasis on their potential therapeutic applications for pain management and for retargeting to non-neuronal tissues. Advances in molecular biology have resulted in generating modified BoNTs with the potential to act in a variety of disorders, however, in addition to the modifications of well characterized toxinotypes, the diversity of the wild type BoNT toxinotypes or subtypes, provides the basis for innovative BoNT-based therapeutics and research tools. This expanding BoNT superfamily forms the foundation for new toxins candidates in a wider range of therapeutic options.

## 1. Introduction

In this review, we summarize the current understanding of Botulinum neurotoxin (BoNT) therapeutic applications together with a special focus on engineering opportunities leading to enhanced therapeutic potential. Nowadays, advances in molecular biology have resulted in generating modified BoNTs with the potential to act in a variety of disorders. In this review, site-directed mutagenesis to modify BoNTs’ binding or substrate cleavage features is discussed in parallel with strategies aimed at retargeting the BoNTs to non-cholinergic neurons or other tissues. The intracellular mechanisms involved in BoNT-induced analgesia are also discussed within the perspective of further engineering to extend their indications related to nociception. It is worth noting that among the 41 naturally occurring BoNTs, only two BoNTs are currently available for therapy, pointing to the prospect of a wide range of unexplored therapeutic opportunities.

BoNTs belong to a family of neurotoxins which are produced by various *Clostridium botulinum* and other atypical strains of *Clostridium* spp., such as *Clostridium butyricum* and *Clostridium baratii*. BoNTs are classified as being part of the “dirty dozen” agents listed as putative bioweapons [1]. Rare but often severe, BoNT intoxication resulting from ingestion of preformed toxin in food or *from C. botulinum* toxi-infection leads to a disease called botulism. Infant botulism and intestinal toxemia in infants above one year and adults are due to the development of *C. botulinum* spores and toxin production in the intestinal tract. More rarely, botulism results from wound contamination. The chief clinical manifestation of botulism is a flaccid peripheral paralysis caused by neurotransmitter release blockade at presynaptic terminals that can be fatal in the absence of intensive care unit support. Botulism treatment is mainly symptomatic including intensive care with mechanical ventilation in the severe cases.

BoNTs (150 kDa) are comprised of a heavy chain (HC, 100 kDa) and a light chain (LC, 50 kDa) [2]. The HC N-terminal domain (HCN, 50 kDa) is the translocation domain, which allows the LC, a metalloprotease, to reach the intracellular compartment [3,4]. In order to enter the neuronal cytosol upon acidification, the LC delivery across the vesicle membrane is facilitated by the translocation domain located on HCN, whereas the HC half C-terminal domain (HCC), which is comprised of two subdomains (HCCn and HCCc) is responsible for specific binding of the toxin to presynaptic membrane of neurons prior to endocytosis [5,6,7,8]. However, the molecular mechanism underlying membrane insertion of HN remains a matter of debate.

BoNTs are produced as botulinum complexes, also called progenitor toxin complexes (PTCs), by non-covalently binding to multiple non-toxic proteins [9]. The PTC non-toxic proteins, referred to as neurotoxin-associated proteins (NAPs) or associated non-toxic proteins (ANTPs), include the non-toxic non-hemagglutinin (NTNH) protein, and either hemagglutinins (HA-17, HA-33, and HA-70) or OrfX and P47 proteins. NTNHs are the major NAPs contributing to toxin stabilization and preservation, while HAs facilitate BoNT absorption during the intoxication process [10,11].

The nine toxinotypes of BoNTs, termed A–G, H or H/A or F/A, and X, cleave one of the three soluble N-ethylmaleimide-sensitive factor attachment protein receptor (SNARE) proteins, i.e., vesicle-associated membrane protein (VAMP), synaptosomal-associated protein 25 (SNAP25), or syntaxin upon entry into synaptic terminals, thereby inhibiting the exocytosis of synaptic vesicles containing neurotransmitters [4]. 

The toxinotype H (also termed H/A or F/A) was identified, in 2014, from a clinical isolate [12]. This toxinotype is composed of a mosaic structure including regions of similarity to toxinotypes A and F with the LC most similar to BoNT/F5 subtype and an HC similar to BoNT/A1-HC [13,14]. Therefore, BoNT/H is neutralized by antibodies against BoNT/A.

BoNT/X has been identified in a *Clostridium botulinum* strain which also synthesizes BoNT/B2 [15]. Moreover, BoNT-like sequences have been found in non-clostridial species such as *Weissella oryzae* and *Chryseobacterium piperi*, BoNT/En protein in an *Enterococcus faecium* strain [16], and paraclostridial mosquitocidal protein 1 (PMP1) in *Paraclostridium bifermentans* strains [17]. The clinical implication or impact of the BoNT-like toxins or sequences are not yet elucidated.

## 2. Overview of Current Therapeutic Applications

The clinical use of BoNTs natural toxins began when Dr. Allen Scott, an ophthalmologist looking for a nonsurgical method, and Dr. Edward Schantz, a microbiologist, started a collaboration to treat overactive muscles [18]. They initially showed that BoNT/A was effective and safe in weakening eye muscles of monkeys and confirmed their findings in humans. The Schantz and Johnson product was subsequently registered under the name Oculinum^®^ through the Oculinum Company, in the late 1970s. Cosmetic application of BoNT/A was recognized accidentally, in 1987, in patients treated for involuntary blinking by ophtalmologists. The Allergan company purchased the company in 1991, renaming the drug Botox to develop its cosmetic uses as Vistabel^®^ and Botox Cosmetic^®^ [19]. Meanwhile, a BoNT product was developed in the United Kingdom by the Public Health Laboratory Service and licensed, in 1992, for Europe under the brand name Dysport^®^ with non-proprietary name abobotulinumtoxinA. The company was acquired by Ipsen France who sold the cosmetic operations to the Galderma company in Switzerland, while the product was renamed Azzalure^®^ [20]. BoNTs are regulatory approved for several disorders related to excessive muscle contractility [21]. BoNTs are classically used in the relief of movement disorders such as in dystonia and spasticity cases [22] but they are also used for the reduction of glandular hypersecretion such as hyperhidrosis or sialorrhea [23]. Furthermore, observations have evidenced the BoNT modulatory role on the sensory feedback loop to the central nervous system (CNS) leading to analgesic effects of BoNT. The toxin has differential effects in excitatory and inhibitory neurons providing a unique therapeutic option in neuronal modulation [24]. Analgesic effects of BoNT have been described in chronic migraines [25]. Currently, BoNTs are widely used in an increasing number of medical conditions associated with muscle spasms, as well as for cosmetic purposes in at least twelve different medical specialties for more than 30 major indications (see Table 1).

The natural BoNTs repertoire already represents an ideal candidate therapy, since a minute amount of this protein is sufficient to silence neuromuscular junctions. In addition to this extreme potency, patients do not classically generate neutralizing antibodies after repeated injections for several years [26]. Moreover, the effects of BoNT/A can last for more than three to six months in humans, since one single injection is sufficient to maintain its therapeutic efficacy for months. Indications that BoNT/A induces skin cell restoration leading to improvement of dermatological conditions suggest a much wider biological influence of BoNTs than the historical SNARE targeting. Recent experimental data have evidenced BoNTs effects on skin flap protection, to facilitate wound healing, ease hypertrophic scars and psoriasiform dermatitis, produce an anti-aging effect, as well as extracutaneous effects such as anti-inflammatory and anticancer properties [27].

The therapeutic use of BoNTs in reducing pain has received full attention, triggering a range of applications in clinical conditions associated with pain. BoNTs inhibit the release of pain-modulating neurotransmitters such as glutamate, substance P (SP), and calcitonin gene-related peptide (CGRP) by synaptic vesicle fusion impairment, and modulation of the transient receptor potential (TRP) of pain-sensing transmembrane receptors at the neuronal plasma membrane [28]. Aoki et al. showed the ability of BoNT/A to inhibit nociception by preventing mechanosensitive ion channel fusion within nerve terminal membrane of peripheral trigemino-vascular neurons. In the context of migraine headaches, the effects of BoNT/A are based on the modulation of the vesicular trafficking, release of neurotransmitters and inflammatory peptides, as well as modification of the expression of relevant ion channels and receptors in neuronal membranes [29]. 

**Table 1:** Timeline for botulinum neurotoxins (BoNT) therapeutic uses.

Year	Author/Institution		Botulinum Toxin, Commercial Designation
**1822**	Justinus Kerner		Sausage poison (first envisioned possible therapeutic use)
**1870**	Müller		Botulism (Latin: *botulus*) for sausage
**1895**	Van Ermengem		*Clostridium botulinum* (causative agent of botulism)
**1919**	G.S. Burke		Determination of minimum lethal dose in guinea pigs
**1928**	Herman Sommer		BoNT (purified) isolation
**1946**	Carl Lamanna Edward Schantz		LD50 test: neurotoxin activity BoNT/A in crystalline form
**1949**	Arnold Burgen		Neuromuscular transmission blockade
**1950**	Vernon Brooks		BoNT/A: blockade of acetylcholine from motor nerve endings
**1960s**	Schantz/Scott		Strabismus: monkeys
**1980**	Scott		Strabismus: humans
**1986**	Joseph Jankovic		Placebo controlled trial of BoNT/A in blepharospasm and cervical-cranial dystonia
**1987**	Drs. Jean and Alastair Carruthers		Cosmetic benefits of BoNT/A found accidentally by ophthalmologists treating patients for involuntary blinking
**1988**	Allergan		Oculinum (BoNT/A): clinical trials
**1993**	Montecucco and Schiavo		SNAP-25, molecular target of botulinum toxin type A
**1995**	MHRA		Approves Dysport^®^ (abobotulinumtoxinA, Ipsen)(~5 ng/500 mouse LD50) for strabismus in UK
**2000**	FDA		Approves Botox^®^ (onabotulinumtoxinA, Allergan) for cervical dystonia
**2001**			Botox^®^ approval for cosmetic procedures in Canada and New Zealand Approves first type B toxin, NeuroBloc^®^ for cervical dystonia
**2002**	FDA		Approves Botox^®^ for cosmetic therapy (Australia, Switzerland, Taiwan, and Singapore)
**2003**	AFSSAPS		Approves Botox as Vistabel^®^ (France)
**2003**	FDA		Approves Myobloc^®^ (rimabotulinumtoxinB, Solstice Neuroscience) for cervical dystonia Neurobloc^®^
**2004**	FDA		Approves Botox^®^ for primary axillary hyperhidrosis (severe underarm sweating)
**2006**	MHRA		Approves Botox as Vistabel^®^ for treatment of glabellar lines
**2006**	-		Xeomin^®^ (incobotulinumtoxinA, Merz) licensed in Germany for blepharospasm and cervical dystonia in adults
**2006**	Korean FDA		Neuronox^®^ (Medy-Tox) approval for blepharospasm
**2009**	MHRA FDA		Approves Azzalure^®^ for treatment of glabellar lines approves Dysport^®^ for glabellar lines and cervical dystonia
**2010**	FDA		Approves Botox^®^ to treat chronic migraine, adult upper limb spasticity, and specific form of urinary incontinence Approves Xeomin^®^ for cervical dystonia and blepharospasm
**2011**	FDA		Approves Xeomin^®^ to treat bladder detrusor over-activity in patients with neurologic conditions
**2011**	FDA		Approves Xeomin^®^ (incobotulinumtoxinA) as Bocouture^®^ for glabellar lines in adult patients
**2012**	NHS UK		Approves Botox^®^ to treat chronic migraine
**2013**	Korea FDA		Approves Nabota^®^ (Daewoongs Pharmaceuticals) approves Botox^®^ for overactive bladder and lateral canthal lines
**2014**	China		BoNT/A product also approved as Lantox^®^ and Prosigne^®^ (Lanzhou Institute of Biological Products, China)
**2015**	FDA		FDA Approval of Xeomin^®^ (incobotulinumtoxinA) and Dysport^®^ (AbobotulinumtoxinA) for adult upper limb spasticity
**2017**			Approval of Botox^®^ and Dysport^®^ to treat adult lower limb spasticity and Dysport^®^ only to treat children lower limb spasticity
**2018**	FDA		FDA approves Xeomin^®^ for sialorrhea Nabota^®^ (Korea 2014) approved by FDA in 2019 Distributed in USA since 2018 as Jeuveau^®^
**2019**	FDA		Approves Botox^®^ for pediatric upper limb spasticityApproves Jeuveau^®^ for glabellar lines

FDA, Food and Drug administration; MHRA, Medicines and Healthcare Product Regulatory Agency; AFSSAPS, French Agency for the Safety of Health products (currently ANSM) (Modified from Rasetti-Escargueil et al. [30] and Whitcup [31].

Given the growing interest in BoNTs’ therapeutic applications, it has become very tempting to enhance the pharmacological properties of BoNTs by increasing their efficacy, moderating their immunogenicity, extending the duration of action or developing fast acting formulations, or targeting specific neurons such as sensory neurons. A longer duration of action would be of great benefit in chronic conditions such as overactive bladder, chronic migraine, or muscle spasticity. Fast-acting formulations would be of great benefit in pain management, as well as for cosmetic applications.

## 3. Exploration of Therapeutic Potential of Novel Toxinotypes or Subtypes and Modified Botulinum neurotoxins (BoNTs)

Harnessing the diversity of the wild type BoNTs toxinotypes or subtypes provides the basis for innovative BoNT-based therapeutics and research tools. Recent progress in sequencing has led to an expanding BoNT superfamily given that this natural repertoire can be explored to form a basis for engineering new toxins candidates in a range of therapeutic options [32]. Successful expression and purification of newly identified subtypes would provide materials for future development of protective vaccines or innovative therapeutic strategies. 

Pioneering work by Lacy et al. established, for the first time, the structural view of BoNT and “brought the toxin molecule to life”. Subsequent structural studies have enabled the correlation between structural differences and distinguished toxin functions [33]. Regarding the structural subdomain organization, despite the high degree of similarity between BoNT/A and BoNT/B, a major difference in BoNT/E domains organization was found by Kumaran et al. [34]. In contrast to BoNT/A and BoNT/B, BoNT/E retains the catalytic and binding domains on the same side of the translocation domain correlating with a more rapid onset of BoNT/E action. In particular, these findings suggest that not all BoNTs toxinotypes share the same fold which has a great impact on the effects of the different BoNT toxinotypes or subtypes opening the way for engineering of improved neurotoxins [35].

The review by Masuyer et al., in 2014 [36] laid the ground work required to design newly engineered BoNTs. BoNTs are modular nano-machines with each domain having its unique function including a binding domain (HCC) that can be employed to target nerve terminals, the translocation domain (HCN) that can be used as a cargo to deliver the LC or a different active protein into cells, and the catalytic LC that can cleave its SNARE substrate which is involved in neuromediator release or cell secretion. Toxinotype-specific characteristics, in terms of kinetics, can be exploited to engineer tailor-made hybrid molecules. The functional LC-HCN fragment can be associated with a retargeting module for the treatment of secretion disorders [36].

### 3.1. Differential Effects of Toxinotypes and New Subtypes

The neuromuscular paralysis, subsequent to a local injection of toxin for the treatment of dystonias, lasts for more than four months with BoNT/A, about two months for BoNT/B, and less than four weeks for BoNT/E, showing that exocytosis of neurotransmitters is blocked for different periods of time depending on the BoNT toxinotypes. The blockade of neurotransmitter release from cerebellar neurons lasts much longer with the type A than with the type B [22,37,38]. BoNT/E and BoNT/F cause only transient blockade of transmitter release that matches with the time of reappearance of intact SNAREs. In humans, BoNT toxinotypes E and F, which are associated with a transient duration of action, can be used in the clinic for short-term treatments such as damaged joint immobilization during pre- and post-operative care. The native BoNT toxinotypes A1 to F1 were compared in ex vivo, in vitro, and in vivo assays. All toxinotypes were found to be highly potent neurotoxins in rodents, except for toxinotype D in vivo. It was found that the differences between different BoNT toxinotypes could be exploited to develop unique and tissue-specific BoNT-based therapeutics. Notably, toxinotypes F1 and C1 were evidenced as potential therapies targeting the somatosensory system, since they showed preference for sensory over motor neurons. In this study, the toxinotype F1 was more potent than B1 and E1 in the (dorsal root ganglia) DRG sensory neurons, but it was the least potent toxinotype in SCN (spinal cord neurons) and CTX (cerebral cortical neurons), suggesting that BoNT/F1 action could favor sensory over motor conditions [39]. 

In clinical settings, it is essential to consider the differences between the toxinotypes duration of action. The rationale for choices between different BoNT toxinotypes becomes interesting for clinicians to obtain a unique combination of efficacy, duration of action, safety, and antigenic potential for each specific preparation. In one recent original study using a range of neurophysiological techniques in healthy subjects receiving various BoNT toxinotypes, BoNT/F induced earlier sprouting and a faster full recovery as compared with any other toxinotypes presenting a slower action profile. Electrophysiological studies characterizing the different toxinotypes would allow physicians to make data-based decisions to select the best toxinotype for each particular patient [40]. It has become desirable to reduce the dose of BoNT injected and prolong the time interval between two administrations, which can be achieved by combining the different properties of each toxinotype in one single hybrid toxin. A new BoNT/A-B hybrid has been constructed that combined the high potency of BoNT/A and high specificity of BoNT/B. This hybrid BoNT may represent an improved treatment option for autonomic disorders [41].

The signature of BoNTs action is their long duration of two to six months in patients particularly for toxinotype A. To highlight this unique feature, the persistence of the BoNT/A subtypes 1 through 5 was determined in primary rat spinal neurons. The persistence of intracellular enzymatic activity of BoNT/A1,/A2,/A4 and/A5 was at least 10 months, while the effects of BoNT/A3 only last for up to five months. The shorter BoNT/E intracellular activity only lasted two to three weeks. This particular longevity of BoNT/A cleaved products could be explored to develop prolonged action formulations [42,43,44]. Conversely, modified BoNT/A1 is designed to achieve shorter persistence of paralysis. The C-terminal domain of BoNT/A1-LC that controls both the onset and duration of the intracellular activity can be mutated in order to create a recombinant BoNT/A1 with different pharmacokinetics in the context of new indications [43].

Analyses of the ten BoNT/A subtypes have revealed different properties, ranging from differential cell entry and enzyme kinetics to different potencies in mice and cell models. The BoNT/A1, 2, 4, and 5 subtypes share a similar duration of action in cultured primary neurons, whereas BoNT/A3 has a significantly shorter duration of action, as shown previously. The local injection of BoNT/A2 in an in vivo model induced faster onset of local paralysis than BoNT/A1, 3, 4, and 5, while BoNT/A3 produced significantly faster recovery of motor-neuron paralysis [45,46].

The short durations of action of BoNT/F and BoNT/E are due to a fast replenishment of synaptobrevin or SNAP-25, whereas the prolonged actions of BoNT/A, BoNT/B, and BoNT/C1 result from the longevity of their respective proteases. A detailed pulse-chase study of native and BoNT-cleaved SNAREs provided seminal evidence of a persistence of BoNT/A protease in the central neurons leading to the prolonged inhibition of neuroexocytosis as opposed to fast replenishment seen with BoNT/F and BoNT/E. These differential durations of action may pave the way for the development of innovative toxin therapies presenting different kinetic profiles to fit with variable therapeutic needs. In addition, the persistence of the light chain due to a particular deubiquitinating enzyme preventing its ubiquitin-dependent degradation represents an opportunity for molecular approaches to reduce morbidity and mortality of BoNT/A [38,40,47,48,49,50].

Further advantages of BoNT variability lie in the various subtypes that largely remain to be explored for their specific properties. The BoNT/A2 represents one of the first main subtypes to be functionally studied. It has been shown to have a faster onset than the current BoNT/A1, as well as a higher potency in vivo and ex vivo models [51,52]. The BoNT/A3 was also found to be less effectively neutralized by anti-BoNT/A1 antibodies, and the symptoms of BoNT/A3 intoxication in mice appeared significantly distinct from those caused by BoNT/A1. It has been suggested that the distinct biological activity arose from structural differences within the binding domains of BoNT/A3 (HCC/A3) and HCC/A1 [53]. The subtle differences of the HCC domains between BoNT/A3 and BoNT/A4 provide a possible explanation of how the structural differences may impact on receptor binding and, subsequently, on different clinical outcomes. Indeed, the changes in the BoNT/A3 and A4 binding domains likely account for their differential binding mode, notably regarding glycan binding specificity [54].

In conclusion, this comprehensive examination of the molecular basis for the extended action of BoNT/A relative to shorter acting toxinotypes in neurons has provided novel information that should aid the extension of therapies, as well as the development of countermeasures for botulism.

### 3.2. Bioengineered BoNTs for Long Duration of Effect

One initial approach to engineer native BoNTs was explored by Dolly et al. [55] who created BoNT-derived enzymatically inactive mutants (BoTIMs) (full-length BoNTs incorporating catalytically inactive LC/A) and combined the BoTIMs with LC/E domains. They obtained one hybrid protein incorporating components of the intracellular persistent LC/A combined with the LC/E and prolonged the duration of action of SNAP-25 cleavage. This prolonged cleavage of SNAP-25 may be beneficial in specific conditions, for example, in the treatment of various pain states, including chronic pain [56,57]. The BoNT/A gene was fused to that of the light chain (LC) of type/E providing a resultant purified protein, LC/E-BoNT/A, that entered cultured sensory neurons and inhibited release of calcitonin gene-related peptide evoked by capsaicin. This engineered LC/E-BoNT/A is one example of a hybrid BoNT that is able to target sensory neurons, including one LC/E more efficient in the blockade of CGRP [55,58].

### 3.3. Bioengineered BoNTs for Increased Activity in Humans

The modulation of BoNT activity can mostly be obtained by mutations of strategic sites of the HC, HCN, and LC [59]. Mutagenesis studies have been classically designed to facilitate the binding of the HC/A and HC/B to the gangliosides and protein receptors to achieve higher efficacy of the toxin. The work by Tao et al. identified mutations of BoNT/B selectively enhancing the binding to human synaptotagmin-II (Syt-II). The neurotransmission blockade by engineered BoNT/B was 11-fold higher than with wild type BoNT/B on cultured neurons [60]. Research has also been carried out to improve the LC activity of BoNT/B on VAMP in vitro [61]. Nevertheless, full-length BoNT/B containing the same mutation did not show enhanced efficacy in cell-based assays or in vivo models [62]. The LC of BoNT/C (LC/C) has also been modified to maintain cleavage of syntaxin 1. This has confirmed the role of syntaxin 1 in synaptic transmission and the therapeutic potential of the BoNT/C [63,64]. Modified rBoNT/B1 toxins showed higher efficacy in human or transgenic mice with improved affinity to hSyt1 and hSyt2. The enhanced affinity to the human receptors induced increased activity in all models that expressed human Syt isoforms. As a consequence, modified rBoNT/B1 toxins showing enhanced affinity to hSyt1 and hSyt2 present an efficient alternative to BoNT/A1 treatments, more specifically for patients who have developed neutralizing antibodies against BoNT/A1 [22,65].

As compared with engineered adaptation of the binding step, modification of the translocation process is more challenging since its molecular mechanism remains poorly understood. The protonation of the surface carboxylates, i.e., glutamate-48, glutamate-653 and aspartate-877 residues, is required for the interaction of the toxin with the negatively charged membranes. Pirazzini et al. [66] identified a triple mutant that showed enhanced activity and faster onset time due to faster cytosolic delivery of the enzymatic domain. Enhanced translocation efficacy by protonation of residues was involved in the translocation of BoNTs. However, the translocation process still remains to be fully elucidated making the engineering based on this process still hazardous [66]. 

More recently, mutagenesis studies have shown the importance of a lipid binding loop for stable binding to membrane-embedded receptors. This has allowed the creation of a more efficient mutant BoNT/B that combined features of a stronger paralytic effect with lower systemic diffusion than the native BoNT/B. Introducing lipid-binding capability to this newly modified BoNT/B offers higher safety and improved therapeutic efficacy [67]. 

Safety and pharmacokinetics of a novel recombinant BoNT/E (rBoNT-E) highlights faster onset of action, greater peak effect at the highest dose tested, and a shorter duration of activity of rBoNT/E as compared with BoNT/A1. This type of treatment has therapeutic potential in patients with spastic muscles, as well potential aesthetic uses [68].

### 3.4. Bioengineered BoNTs for Targeting Sensory Neurons and Treatment of Pain

In contrast to tetanus toxins, BoNTs were postulated to remain localized at the injection site. However, BoNTs in vivo axonal retrograde transport and transcytosis across neurons were established by Caleo’s group [69,70,71,72,73,74,75]. Therefore, a more comprehensive understanding of the trafficking to the central nervous system (CNS) and central actions of BoNTs is required, in particular, for their therapeutic applications to human central neurological diseases such as epilepsy and pain management [76,77,78,79,80,81,82].

The first indications of pain relief effects of BoNT emerged from patient’s reports upon dystonia treatment. Treatment with BoNT put an end to the vicious circle of muscle spasm/pain, helping the return to normal activities and, subsequent, long-term recovery [83,84,85,86].

In addition to naive toxins, BoNTs “look alike” that were produced by retargeting of recombinant chimera to nociceptive neurons or by “protein-stapling” technology of re-assembled BoNT from two separate fragments are opening new opportunities for chronic pain management using safer and more precisely targeted neuronal silencing agent [87,88] (Table 2). 

The proof of principle study by Wang et al. [89] showed that new engineered toxins could be tailored for specific applications by targeting specific populations of neurons or secretory cells. The LC/E-BoNT/A chimera was bound successfully to sensory neurons and blocked pain peptide release, while BoNT/E alone was unable to bind to sensory neurons [89]. A more recent advance has been the successful engineering of a chimera from two BoNTs to acquire the capability of targeting sensory neurons, and thus inhibiting the release of pain mediators. This novel recombinant protein (BoTIM) blocks the exocytotic response triggered by a stimulant of nociceptive C fibers [55,90,91,92]. The capability of retargeting a more active moiety to sensory neurons, leading to inhibition of pain mediator release, suggests potential applications of recombinant BoNTs in a variety of chronic pain conditions that do not respond to existing drugs [93,94,95]. Indeed, LC/E-BoNT/A abolishes the TNFa-dependent surface trafficking of TRPV1/A1 channels involved in pain sensation. As TNFa induces nociceptive hypersensitivity in vivo, the inhibition by LC/E-BoNT/A of its effect on increased membrane expression of nociceptors in cultured sensory neurons could contribute to alleviation of pain [96].

In addition to the re-engineering of BoNT, a new “protein-stapling” technology has recently been developed that re-assemblied BoNT/A from two separate fragments. This new technology safely produced a less paralytic version of a neuronal silencing agent. The re-assembled toxin exhibited a potent inhibition of CNS function but with no systemic toxicity after intra-peritoneal injection. This unique technology also represents a new tool for the development of safer neuronal modulating agents needed in neuroscience research and medical applications. The stapled chimera inhibited mechanical hypersensitivity in a rat model of inflammatory pain and blocked neuronal activity in a defined area of visual cortex without causing either flaccid or spastic paralysis. This provided the first evidence that the protein stapling technology could assemble distinct proteins yielding new biomedical properties. Thanks to novel functional characteristics, this re-assembled neurotoxin can be used for silencing CNS neurons without causing generalized paralysis [87].

Recently, one study investigated the activity of vector-expressed transgenic BoNT/LC, in cultured sensory neurons. Silencing effects of transgenic BoNT/LCs was shown on sensory neurons. The very large genomic capacity of herpes vectors conferred long-term potential of transgenic expression, and selective or controllable BoNT/LC expression in different types of neurons, by introducing BoNT/LC elements under the control of neuron-specific promoters. Engineering of BoNT active domains into viral vectors opens new perspectives both for understanding the basic biology of BoNTs, as well as for the development of novel therapeutic indication [97]. 

Tang et al. demonstrated an efficient and robust sortase-based method for ligation of recombinant BoNT enzymatic and translocation domains (LC-HCN) called core-therapeutics to ligands targeting the delivery of SNARE-cleaving protease into specific cell types. This targeted delivery of a SNARE protease to specific neuronal subtypes or non-neuronal cells represents a revolutionary approach for specific BoNT applications such as pain relief. These novel recombinant BoNTs, linked to specific cell type ligands that are able to inhibit the release of pro-inflammatory cytokines or major pain neuropeptides, are promising candidates for in vivo studies in animal models of inflammatory and neuropathic pain [88].

**Table 2:** BoNTs engineering applications.

BoNT	Modification	Application	Reference
BoNTs	Re-engineering of target specificity	Chronic pain	[98]
BoTIMs	Full-length BoNTs incorporation inactive LC/A and LC/E	Prolonged effect in various pain states including chronic pain	[55,90,99]
BoNT/B_MY_	Mutations enhancing binding to human synaptotagmin-II, mutations of the lipid binding loop	Enhanced efficacy	[60,65,67,100]
LC/B	Mutations of substrate recognition pockets	Novel therapy to escape immunoresistance in BoNT/B therapy.	[61]
BoNT/LC	LC Mutations	Maintain cleavage of syntaxin	[64,101]
BoNT/B TM (triple mutant)	Mutations inducing protonation of residues involved in translocation process	Increased neurotoxicity due to faster cytosolic delivery of the enzymatic domain	[66]
BoNT/A	Protein stapling allowing BoNT/A re-assembly in situ	Development of neuronal modulating agents	[87]
BoNT/A and E chimera	Chimera construction	Targeting specific populations of neurons or secretory cells	[89]
BoNT/LC	Vector-expressed transgenic BoNT/LC	Stable, selective, and controllable, BoNT/LC expression in different neuron types	[97]
BoNTs	Ligation to agents targeting BoNT delivery into specific cell types	Pain relief, inflammation and neuropathic pain	[88]

## 4. Harnessing BoNTs to Retarget Non-Neuronal Territories

BoNT is one of the most potent and deadliest substances on earth. Because of its unique ability to precisely deliver the toxin locally, the toxin is used for effective treatment of a wide range of diseases including migraine and muscle spasticity. But this unique ability to deliver and act locally can be exploited to target other territories, thanks to molecular engineering techniques. In addition, this ability to deliver the drug locally and minimize systemic exposure is key to target peripheral neurons and address experimental questions about neural physiology or restorative therapies. Engineering of recombinant functional BoNTs is also essential in the characterization of the novel BoNT-like sequences that have been identified in non-clostridial species but that are not known to synthesize functional toxins [102].

BoNT retargeting is facilitated by the modular structure-function of BoNTs. Indeed, the BoNT/A fragment encompassing the LC and HN domains (LC-HN) and without the receptor-binding domain is unable to bind to neuronal cells, but the remanence of the translocation domain (HN) allows the formation of pores in membranes under acidic conditions [103]. The crystal structure of LC-HN/A reveals a preserved structural conformation even with the absence of the HC domain [104,105,106]. These structural properties make the LC-HN fragment an efficient delivering system of LC into cell types not naturally targeted by BoNTs [22]. Initial work using this approach involved the use of a LC-HN/A preparation chemically coupled to lectin wheat germ agglutinin and nerve growth factor (NGF). The resulting conjugate was able to inhibit the release of noradrenaline from the catecholamine secreting PC12 cells [107]. These initial studies showed that the BoNT endopeptidase might be successfully delivered into a range of distinct neuronal and non-neuronal cell types.

**Figure 1:**
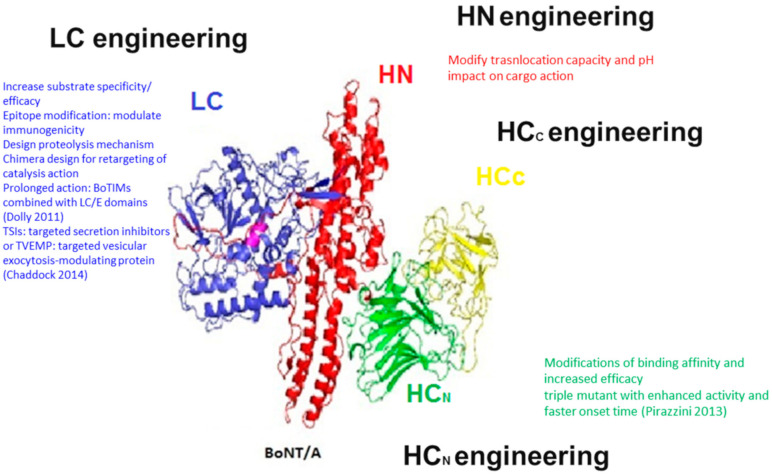
Summary of the BoNTs engineering opportunities.

On the basis of the unique properties of the BoNTs, a novel class of proteins was created (targeted secretion inhibitors (TSIs) or targeted vesicular exocytosis-modulating protein (TVEMP)) that is comprised of three basic domains, each contributing to the function of the whole molecule (Figure 1). The LC domain of one selected BoNT toxinotype confers SNARE cleavage capability depending on selected BoNT toxinotype. The HN domain provides the intracellular translocation ability for the LC and the binding domain may be derived from BoNT but is more often a peptide or a protein that interacts with a receptor of choice on the target cell. This innovative approach exploits the endopeptidase domain for the purpose of modulating the intracellular processes of the target cells and inhibiting their secretion mechanisms [35,108,109]. 

The more recent studies using the recombinant LC-HN fragment expressed in *E. coli* have greatly improved the fusion of proteins combining LC-HN with a targeting entity [110]. In addition to this approach, exploitation of the toxinotype variability allows an extension to the cleavage of a wide range of SNARE proteins. This can open new therapeutic avenues by targeting non-neuronal cell types that do not specifically express SNAP-25. Using existing co-crystal structure, molecular dynamics and mutagenesis studies have made it possible to engineer novel BoNTs for the treatment of SNAP-23-mediated hypersecretion disease. These BoNT-based secretion inhibitors can deliver the toxin to a specific cell type to inhibit vesicular secretion forming the basis for engineering novel secretion inhibitors. The potential of such molecules for the treatment of asthma or chronic obstructive pulmonary disease was evidenced with the use of LC-HN/C linked to epidermal growth factor (LC-HN/C-EGF) inhibiting the release of mucin by pulmonary epithelial A549 cells (COPD) [93]. Understanding the structure–function relationship of BoNTs with their substrates has helped to overcome the SNAP-25 substrate limitations by extending the area where BoNTs exercise their action. Thereby, engineered BoNT/E LC extends the cleavage spectrum of wild type BoNT/E from human SNAP-25 to SNAP-23 [35,111,112]. In addiiton, engineered BoNT/A was generated to cleave SNAP-23, expanding the use of BoNT to non-cholinergic-dependent applications [113]. SNAP-23 regulates the secretion in numerous cells such as that of pro-inflammatory factors. It is noteworthy that albeit wild type BoNT/A does not cleave SNAP-23, this toxinotype downregulates the expression of SNAP-23 and production of pro-inflammatory factors, and thus attenuates neuropathic pain [114].

Chen et al. demonstrated the inhibition of interleukin-8 (IL-8) and mucin release from stimulated HeLa cells by extending the substrate specificity of BoNT. This proof of concept, showing that an engineered LC of toxinotype E can impair vesicle trafficking when directly delivered into cultured human epithelial cells, widely expands the therapeutic potential of the BoNTs for non-neuronal human secretory diseases [111]. 

In addition to the targeting of different SNARE targets, engineered BoNT can be exploited to deliver other molecules into neurons (Table 3). Most biological toxins target specific cell types by delivering their enzymatic domains into the cytosol. The toxin delivery process can be exploited by engineering of chimeric toxins. The *C. botulinum* C2, which destabilizes the actin cytoskeleton in various cell types, was retargeted to neural cell populations by deleting its non-specific binding domain and replacing it with a BoNT binding domain. The retargeted toxin enables delivery of compounds including therapeutic drugs to peripheral neurons and addresses experimental questions about neural physiology [115]. A similar approach was successfully implemented to bioengineer BoNT by removing toxicity without disrupting neuron-specific targeting, thereby creating a molecular vehicle capable of delivering therapeutic cargo into the neuronal cytosol. This study established that engineering of BoNT/C1 could be useful as a molecular vehicle for drug delivery to the neuronal cytoplasm [116]. 

## 5. Production of Inactive BoNT Holoprotein for Vaccines Development

Beyond the creation of innovative retargeted proteins, engineering of the BoNTs has resulted in the production of safe vaccine candidates (Table 4). While the intact BoNT proteins are lethal, independent domain-length fragments are devoid of toxicity when injected into animals [118,119]. Advances in genetic engineering have resulted in the production of highly purified recombinant protein antigens representing one or more BoNT domains. The novel possibility of generating specific genetic sequences and producing purified recombinant proteins into expression platforms would enable the development of vaccine antigens that retain the immunogenicity of the parent BoNT, without their lethality. There are currently nine BoNT toxinotypes, denoted A–X, that diverge by 37.2–69.6% at the amino acid level, thus, requiring seven toxinotype-specific vaccines for protection against all BoNT types. In addition, each toxinotype contains subtypes which differ in amino acid sequences from 1.6 to 36.2% [32,120]. This variability influences protection with neutralizing antibodies impeding the development of prophylactic tools against a wide array of BoNT subtypes [121].

Recombinant subunit vaccines based on BoNT HC domain against toxinotypes A–F have been demonstrated to be safe and effective. BoNT HC vaccines for all nine toxinotypes, based on sequences from subtypes (A1, B1, C1, D/C, E1, F1, and G), have been developed including a recombinant botulinum vaccine for BoNT toxinotypes A and B (rBV A/B), tested in monovalent, bivalent, and trivalent formats and validated as highly effective vaccines, safe and easy to produce [122,123,124,125,126,127,128]. However, to meet the BoNT subtypes variability showing significant amino acid heterogeneity, catalytically inactive BoNTs (ciBoNT HPs) have been developed to achieve greater protective immunity against the numerous BoNT variants [126]. One recombinant, catalytically inactive BoNT/A1 vaccine (ciBoNT/A1 HP) was originally produced in 2009 showing greater potency than any recombinant protein encompassing A1 subtype domain, or combination of domains [129]. This research was expanded with the production of recombinant ciBoNT/B1 HP, ciBoNT/C1 HP, ciBoNT/E1 HP, and ciBoNT/F1 HP protein antigens. The ciBoNT HP vaccine antigens elicited a more robust neutralizing antibody response and better protection against a challenge from the parental toxins and superior protection against challenges from dissimilar subtypes [126]. The benefit of the recombinant ciBoNT HP vaccine antigens are due to the combination of the three protein domains expanding the diversity of neutralizing epitopes as compared with the individual subunit vaccines.

**Table 4:** Recombinant BoNTs for vaccination.

BoNT Sub-Unit	Applications	References
LH_N_ fragments from BoNT/A and B	Single-dose protection against BoNT/A1, A2, and A3 and against BoNT/B1and B4 (nonproteolytic)	[118]
rBV A/B recombinantly derived from the non-toxic C-terminal domains of BoNT/A1 and BoNT/B1	Protection against BoNT/A1 and BoNT/B1	[119]
Recombinant BoNT A HC, BoNT B HC, BoNT C HC, BoNT D HC,) BoNT E HC, and BoNT F HC produced in *Pichia pastoris*	Protection against BoNT/A, B, C, D, E, and F respectively	[122,125,130,131,132,133,134,135,136]
ciBoNT/A1 HP ciBoNT HPs	Protective immunity against the BoNTs variants	[126,137]
BoNT/A1 LC–HN	Protection against BoNT/A1, A2, and A3	[129]
BoNT/A1 LC–HN+HC	Protection against BoNT/A	[138]
Multivalent HC/A, HC/B, and HC/E vaccine	Protection against BoNT/A, B, and E	[127]

## 6. Future Approaches and Perspectives

In 1822, Justinus Kerner first envisioned the potential therapeutic uses of the “fatty acid agents” that blocked the parasympathic drive in animals, later named “*Clostridium botulinum*” by Dr Van Ermengen. Since then, the BoNT was isolated and purified at the beginning of the 20th century and its therapeutic utility was initially confirmed in strabismus. While its structure and modular architecture were explored and elucidated, the therapeutic indications have been increasing until today to reach a wide spectrum of indications ranging from dystonia, hyperhidrosis, urological disorders, migraines, and cosmetic uses.

The modular structure of the toxin has driven the many opportunities to engineer the BoNTs into more efficient, less toxic or retargeted agents active at non-neuronal territories. Nevertheless, gaining even more insights into BoNT engineering to extend therapeutic interventions related to nociception will help the development of new ways to alleviate acute or chronic human pain. Currently in the exploratory phase, there are also major expectations of advances in neuroprotective strategies, neuronal burgeoning, treatment of hypersecretory, and hormonal disorders by engineered BoNTs [139].

Significant progress has been made on the creation of atoxic BoNT fragments for the design of vaccines. However, the wide natural repertoire of BoNT toxinotypes and subtypes remains to be a challenge in the development of efficient countermeasures. In the future, another important aspect is optogenetic techniques for local manipulations of photoactivatable BoNTs, leading to inducible control of neurotransmission. 

## Figures and Tables

**Table 3:** BoNTs retargeting methods.

BoNT	Modification	Application	Reference
LC-HN fragment	Targeted delivery	Deliver LC into cells not naturally targeted by BoNT	[100]
LC-HN/A	Coupling to lectin wheat germ agglutinin	Inhibit noradrenaline release	[107]
TSIs: targeted secretion inhibitors or TVEMP	LC domain (SNARE cleavage capability) HN domain (intracellular translocation) binding domain a peptide interacting with target cell	Treatment of pain, endocrine disease (acromegaly) and cancer	[35,110]
LC-HN part of BoNT	LC-HN coupled to epidermal growth factor (LC-HN-EGF)		[96,117]
BoNT/E LC	Mutations	Cleave human SNAP-23 for treatment of asthma or hypersecretions	[112]
BoNT/A	Protein stapling technology	Neuroscience research and future medical applications in chronic pain	[87]
BoNT/E	Mutation of LC	inhibition of interleukin-8 (IL-8) and mucin release	[111]
BoNT/C	Mutations of C2 binding/translocation domain	delivery of therapeutics to peripheral neurons	[115]
